# Example-based learning: comparing the effects of additionally providing three different integrative learning activities on physiotherapy intervention knowledge

**DOI:** 10.1186/s12909-015-0308-3

**Published:** 2015-03-07

**Authors:** Joseph-Omer Dyer, Anne Hudon, Katherine Montpetit-Tourangeau, Bernard Charlin, Sílvia Mamede, Tamara van Gog

**Affiliations:** 1 School of Rehabilitation, Faculty of Medicine, Université de Montréal, P.O. Box 6128, Station Centre-Ville, Montreal, QC H3C 3J7 Canada; 2 Centre de pédagogie appliquée aux sciences de la santé (CPASS), Université de Montréal, Montréal, QC Canada; 3 Centre for Interdisciplinary Research in Rehabilitation of Greater Montreal (CRIR), Montreal, QC Canada; 4 Department of Neurology, Montreal General Hospital, Montreal, QC Canada; 5 Institute of Medical Education Research Rotterdam, Erasmus Medical Center, Rotterdam, The Netherlands; 6 Department of Psychology, Erasmus University Rotterdam, Rotterdam, The Netherlands

**Keywords:** Clinical reasoning, Self-explanation, Concept map, Worked example, Completion example, Physiotherapy

## Abstract

**Background:**

Example-based learning using worked examples can foster clinical reasoning. Worked examples are instructional tools that learners can use to study the steps needed to solve a problem. Studying worked examples paired with completion examples promotes acquisition of problem-solving skills more than studying worked examples alone. Completion examples are worked examples in which some of the solution steps remain unsolved for learners to complete. Providing learners engaged in example-based learning with self-explanation prompts has been shown to foster increased meaningful learning compared to providing no self-explanation prompts. Concept mapping and concept map study are other instructional activities known to promote meaningful learning. This study compares the effects of self-explaining, completing a concept map and studying a concept map on conceptual knowledge and problem-solving skills among novice learners engaged in example-based learning.

**Methods:**

Ninety-one physiotherapy students were randomized into three conditions. They performed a pre-test and a post-test to evaluate their gains in conceptual knowledge and problem-solving skills (transfer performance) in intervention selection. They studied three pairs of worked/completion examples in a digital learning environment. Worked examples consisted of a written reasoning process for selecting an optimal physiotherapy intervention for a patient. The completion examples were partially worked out, with the last few problem-solving steps left blank for students to complete. The students then had to engage in additional self-explanation, concept map completion or model concept map study in order to synthesize and deepen their knowledge of the key concepts and problem-solving steps.

**Results:**

Pre-test performance did not differ among conditions. Post-test conceptual knowledge was higher (*P* < .001) in the concept map study condition (68.8 ± 21.8%) compared to the concept map completion (52.8 ± 17.0%) and self-explanation (52.2 ± 21.7%) conditions. Post-test problem-solving performance was higher (*P* < .05) in the self-explanation (63.2 ± 16.0%) condition compared to the concept map study (53.3 ± 16.4%) and concept map completion (51.0 ± 13.6%) conditions. Students in the self-explanation condition also invested less mental effort in the post-test.

**Conclusions:**

Studying model concept maps led to greater conceptual knowledge, whereas self-explanation led to higher transfer performance. Self-explanation and concept map study can be combined with worked example and completion example strategies to foster intervention selection.

**Electronic supplementary material:**

The online version of this article (doi:10.1186/s12909-015-0308-3) contains supplementary material, which is available to authorized users.

## Background

Clinical reasoning is a decision-making process that allows clinicians to determine the correct diagnosis and select the most appropriate intervention for a clinical problem [1,2]. Developing clinical reasoning skills is quite a challenge for novice learners since these abilities are, in part, based on tacit knowledge gained through clinical experience [3]. Example-based learning is an instructional method that can help foster clinical reasoning by providing learners with examples of clinical cases and their management [4,5]. Example-based learning has been shown to be more effective when learners self-explain the material being learned [6]. Adding prompts to induce self-explanations also fosters acquisition of problem-solving skills from faded worked examples, that is, when learners first study a fully worked-out example and then complete steps in partially worked-out examples [7]. Self-explanation is thought to help learners achieve more meaningful learning by deepening their understanding of the information presented. Constructing concept maps (i.e., concept mapping) and studying concept maps (i.e., concept map study) are other instructional activities that can promote meaningful learning by engaging learners in reorganizing their knowledge. However, concept maps have received little attention in combination with example-based learning. In health profession education, concept maps are often used in association with other instructional methods as additional integrative activities [8]. This raises the question as to which of these three integrative learning activities (i.e., self-explaining, concept mapping, or concept map study) would be best for promoting meaningful learning in addition to example-based learning among physiotherapy students.

### Example-based learning

Example-based learning integrates the theoretical principles of three research areas in education: analogical reasoning, observational learning, and learning from worked examples [9]. Common principles derived from these research areas emphasize the importance of: a) learning with examples of a particular case, b) providing multiple examples, c) linking examples to underlying principles and d) prompting learners to make connections between examples and principles [9]. The analogical reasoning theory stipulates that providing relevant examples of cases showing the process of problem-solving can enable learners to make useful analogical inferences when confronted with new problems [10]. Clinicians usually rely on previous problem-solving experience to make these analogical inferences [11]. Novice learners, who lack experience, can study examples of cases to develop their own bank of relevant cases on which they can rely to solve new problems [10]. Some principles of example-based learning are also based on observational learning [12]. This type of learning occurs when a person gains knowledge by observing a modelling example in which another person (i.e., a model) performs a task [13]. More specifically, when educators have to deal with the acquisition of cognitive skills based on underlying abstract principles, *abstract modeling* can be used to allow learners to study a modelling example in which the model explains his reasoning [9]. In example-based learning with worked examples, learners have to study a didactical problem-solving solution that presents the way in which they should learn to manage a specific problem [14,15].

In health profession education, example-based learning provides an opportunity to put into practice several relevant strategies for fostering clinical reasoning. These strategies mainly consist of improving learners’ knowledge, data gathering, data analysis, and metacognition [16]. In terms of improving learners’ knowledge, educators must take into account that organizing knowledge into a useful way is more important than the amount of knowledge stored in memory [17]. Clinicians organize, memorize and access their knowledge using cognitive structures known as illness scripts in order to efficiently recognize relevant features and interpret and manage a clinical problem [18,19]. Scaffolding strategies that can help learners develop their own illness scripts provide a sound basis for fostering clinical reasoning [16]. Scaffolding involves assisting learners in understanding concepts, solving problems or achieving goals that would have been beyond their abilities without guidance [20]. Example-based learning is a form of scaffolding that can improve learners’ knowledge by presenting the key features and solution steps of a clinical problem in a structured fashion to help learners acquire as well as organize new knowledge in relation to prior knowledge. Providing learners with case examples before they can actually solve problems by themselves can be a part of a scaffolding-based learning strategy. These scaffolds can be gradually faded as learners acquire more knowledge by presenting them with completion problems after worked examples, for which learners have to fill out missing steps in partially worked-out examples [7].

Example-based learning can not only help students acquire illness scripts, but also foster clinical reasoning by improving data gathering and processing. Data gathering skills include collecting relevant data and performing appropriate physical examinations [21]. Learners develop these skills by studying non-didactical examples based on the actual performance of expert models managing clinical cases [4]. Data processing aims to abstract the clinical information into a meaningful patient assessment and plan of care [16,22]. Coaching problem representation via the use of semantic qualifiers, in which the content of an observation is given an abstract form along oppositional relationships [23,24], may help in comparing hypotheses and acquiring data processing skills [16]. Moreover, example-based learning can improve metacognition. Metacognition refers to the ability of recognizing one's own cognitive processing [25]. Reflection is a metacognitive strategy that can be useful for improving clinical reasoning [26-29]. Facilitating learners’ reflection using prompts or questions to make them deliberately connect key elements of a clinical case with an illness script can help learners identify learning points for application in similar cases in the future [16]. Such teaching strategies can potentially help learners by making it easier to activate appropriate illness scripts that will help them solve similar cases [16,18]. However, it is still unclear how example-based learning activities should be designed to optimize the acquisition of problem-solving skills. In science education, learning activities based on problem solving typically include both worked examples (where the problem-solving procedure is worked out step by step) and practice problems (where no instructional guidance is given) [14]. It has been reported that such combinations of examples and problems can foster diagnostic reasoning in medicine [30]. Some evidence from other domains suggests that novice learners rely heavily on examples to solve problems presented to them [31]. Furthermore, it has been demonstrated that practicing with examples/problem pairs is an efficient way to foster problem-solving skills [14,32-34]. In these studies, each example is followed by a comparable yet not identical practice problem [14,33,34].

### Worked examples and completion examples

A worked example is an educational tool that provides learners with a fully worked-out solution to a problem, which they can study to build a cognitive schema on how to solve such problems [35]. Worked examples are therefore mainly effective in the initial stages of knowledge acquisition [36]. They have been successfully used in fields with well-defined problems such as mathematics and physics [37] but also in areas with ill-structured problems such as health professions. Ill-structured problems are complex problems in which the initial presentation does not necessary provide all the information required to develop a solution. Moreover ill-structured problems can be solved in more than one correct way [18]. One should consider designing process-oriented worked examples to foster complex cognitive skills [38] such as those required for solving ill-structured problems. Process-oriented worked examples include information pertaining to principles (“why”) and strategies (“how”) that experts use when solving problems [38]. Process-oriented worked examples have been used to facilitate the collaborative diagnosis of a patient in medicine and psychology [39], and history taking in physiotherapy [4]. Worked examples presented in a digital learning environment can foster diagnostic reasoning among medical students [40-42]. The learning effect of worked examples in novice learners is superior compared to direct teaching of problem-solving principles or activities [43,44]. This effect can be explained by cognitive load theory (CLT), which states that effective instructions should take into account the limitations of human working memory [45]. CLT distinguishes between three distinct and additive types of load that can be imposed on working memory during learning: extraneous load, intrinsic load and germane load. Extraneous load originates from element interactivity due to the design of the instructional activity and does not contribute to learning; intrinsic load refers to the inherent difficulty of the task being learned and is determined by the number of interacting elements in the task itself; germane load arises from cognitive processes associated with effective learning and may be triggered by instructional activity [46]. Recent discussions on CLT have proposed to revisit this model by considering only the first two types of load (i.e., extraneous and intrinsic load) because germane load is essentially indistinguishable from intrinsic load [47]. In accordance with this new proposition, the theoretical concept of germane load is thought to be useful only if it is redefined as referring to the actual working memory resources devoted to dealing with intrinsic load [47]. Because novice learners have not yet acquired effective problem-solving strategies, they have to rely on inefficient strategies which impose high extraneous working memory load but leads to learning only very slowly. Compared to problem-solving activities, worked examples facilitate learning by decreasing extraneous load associated with ineffective strategy use, and simultaneously allow for germane processes associated with schema construction [34]. Not surprisingly, therefore, a substantial body of evidence demonstrates that pairing worked examples with practice problems is a more effective strategy for improving problem-solving skills than presenting learners solely with practice problems [12,14,34,48].

It is worthwhile combining examples with problem-solving activities. Studying worked examples alone does not guarantee that learners will effectively use the available working memory resources for learning. A potential drawback of worked examples, especially in authentic educational settings, is that learners may simply glance over the examples and not study them in enough detail. When considering this issue, providing additional instructions or activities to ensure that learners are more actively engaged in studying worked examples might be a relevant strategy for improving learning. One strategy to ensure that learners pay enough attention to worked examples is to provide them with completion examples [49,50]. As mentioned above, a completion example is a worked example in which learners must complete some key solution steps of the problem [51]. Learners who use completion examples perform better on problem-solving tasks than those who learn with a trial-and-error-based strategy [50,51]. Moreover, the fading strategy in which learners must gradually complete more steps of the example may serve as an alternative method to studying fully worked-out examples and problem solving [30,52,53]. This strategy was found to be more effective for fostering problem-solving skills than example/problem pairs, especially backward fading in which the last solution steps are omitted from the completion examples [30,35]. In short, example-based learning with worked/completion example pairs involving backward fading is an efficient learning strategy for fostering meaningful learning of problem-solving skills in novice learners.

### Meaningful learning to foster clinical reasoning

Meaningful learning to foster clinical reasoning occurs when learners develop cognitive skills that allow them to integrate new information with existing knowledge in order to efficiently solve new clinical problems [54,55]. The organization of clinical knowledge is crucial for the development of clinical reasoning [17]. Clinical knowledge includes factual, conceptual and procedural knowledge [56]. Factual knowledge reflects knowing facts without in-depth understanding, and its acquisition alone would not necessarily lead to an improvement in clinical decision-making performance [56]. Conceptual knowledge refers to the understanding of principles governing a field of interest and the interrelations between units of knowledge [57]. Improvement in conceptual knowledge can benefit decision making [56]. Procedural knowledge reflects knowing how to perform an activity [56] and is in part implicit [58]. Procedural knowledge is involved in clinical-problem solving [59] and includes strategic and teleological knowledge, as well as heuristics [38,56]. Strategic knowledge involves knowing strategies and rules underlying decision-making for solving problems in a specific domain. Teleological knowledge involves understanding the rationale behind procedure and solution steps. Heuristics is defined as simple, and often unconscious, decision-making strategies that use less than complete information [60]. Heuristics can influence clinical-decision making performance in both positive and negative ways [61]. To achieve meaningful learning, learners need to understand the rationale and context of application of the problem-solving strategies being learned [38,40]. Learning activities that facilitate the acquisition of conceptual and procedural knowledge might foster meaningful learning of decision-making skills. It is unclear how instructional activities should be designed to optimize learning of conceptual and procedural knowledge that is relevant for solving ill-structured problems in health professions. A simple approach to achieving meaningful learning with worked/completion example pairs would be to add a subsequent integrative metacognitive activity in order to deepen learning without interfering with the worked/completion example effect. Learners are typically instructed to reflect by themselves on what they should remember from the lesson to promote in-depth learning [62]. This is often achieved by using prompts and questions to facilitate self-explanations on the learning material [35]. Concept mapping and concept map study are two other integrative activities that could be additionally provided in example-based learning to foster more meaningful learning by helping learners’ organizing their own knowledge.

### Self-explanation

Self-explanation can be defined as the action of generating explanations for oneself in order to make sense of new information [63]. This metacognitive strategy fosters meaningful learning by triggering key cognitive processes. These include: 1) generating inferences from the material presented; 2) making links between information; 3) integrating new information into prior existing knowledge; and 4) reorganizing one’s knowledge representation [63,64]. Some studies have investigated the use of self-explanation in combination with direct instruction methods [65]. These studies differ in whether self-explanation is promoted by training or a prompting intervention [9]. The training approach mainly consists of providing learners with: a) information about the importance of self-explanation, b) a model of self-explanation, and c) a coached self-explanation practice [9]. Such self-explanation training was found to be successful for promoting learning in various domains and instructional setups [62,66,67]. In addition, digital learning environments provide an opportunity to promote self-explanation by using prompts that can be strategically presented in order to focus learners’ attention on key aspects of learning material [68,69]. In these computer-based environments, learners are usually instructed to type their self-explanations into text boxes [9]. Some evidence suggests that typical prompts that worked well ask learners for overall principles that can be applied for problem-solving [7,70]. Using prompts to promote self-explanation leads to better transfer than studying without prompts [35,66]. Self-explaining can benefit conceptual knowledge and promote transfer in various fields [57,65,66,71]. Transfer refers to the learner’s ability to use knowledge gained from an instructional event to solve subsequent problems [72]. Moreover, one can distinguish between near and far transfer skills. Near transfer refers to the ability to solve new problems that have a similar structure (same solution rationale) but different surface features (not relevant to problem-solving) than problems previously studied [30]. Far transfer is the ability to solve new problems with dissimilar structure and surface structure than those previously studied [30]. Some evidence suggests that self-explanation may be a relevant activity for promoting transfer in health education professions. Among medical students, self-explanation can improve diagnostic performance when confronted with less familiar topics [64]. Repeated self-explanation without prior training in the use of this method can improve retention and knowledge application among medical students about 6 months after the initial training session [73]. The key aspect of the self-explanation effect is that learners attempt to revise their understanding and make sense of the material, even if they are unsuccessful in articulating a correct explanation [35,74]. For learners engaged in backward fading with worked/completion example pairs, presenting simple self-explanation prompts in the form of simple questions on some solution steps increases near transfer performance compared to backward fading without prompting [7].

### Concept maps

Concept mapping is another tool that can be used to foster meaningful learning by facilitating the development of self-assessment and individual thinking processes [75-77]. Concept maps are graphic representations of organized knowledge [78]. They consist of concepts represented in cells or nodes that are cross-linked by lines or arrows in order to explain the relationships between the concepts. In hierarchical concept maps, the broadest or most general concepts are presented at the top or in the middle of the map and the more specific sub-concepts are presented below or off to the side [78]. Using a question as the primary concept is recommended in order to keep the focus on the main idea developed in the map. Concept mapping allows learners to organize and present their knowledge in order to develop meaningful learning [79]. Having learners make concept maps is widely used as an instructional tool in health professions [8]. Concept maps can be useful in learning complex knowledge since it involves active information processing and organization of information within a hierarchical framework [78]. To construct a hierarchical concept map, learners must assess the relative inclusivity and specificity of concepts and also process the information in order to subsume lower-order concepts under high-order concepts [80,81]. Moreover, learners engaged in concept mapping must also use a process of integrative reconciliation to make links between differentiated concepts [77,81]. Although these processes can be cognitively demanding, some evidence shows that effective learning occurs in learners engaged in this activity. Concept mapping is more effective in promoting knowledge retention and transfer than reading text or attending lectures [82,83]. Concept mapping has been used to develop critical thinking in nursing students [76,77,84]. Some evidence suggests that learning activities involving concept mapping produce better learning than traditional courses among medical students [54].

However, making a concept map is likely to require considerable working memory capacity. Concept mapping will only aid learning if there is sufficient capacity available, which may not always be the case when learners are working on authentic, complex cases. In this situation, model concept maps can be provided to learners, to help them organize and integrate their knowledge. When compared to text reading, studying concept maps may lower cognitive load by organizing concepts to represent relatedness and explicitly labelling links to show relationships [83]. Concept map study may also foster deep learning by encouraging learners to judge the importance of the concepts and process their hierarchical organization and the relationships between them [80]. Studying concept maps can facilitate recall of the main ideas presented in the map [85]. There is a body of evidence showing that concept maps study produces better knowledge retention than studying text passages, lists or outlines [83,86-89]. However, there is insufficient evidence that model concept map study can promote knowledge transfer [83]. Studying and completing didactical concept maps related to short clinical cases did help develop meaningful learning in medical students [90]. Studying an expert concept map as an advanced organizer was found to improve knowledge integration and deepen the understanding of medical knowledge in resident physicians [91].

Given this evidence, one can hypothesize that adding these activities to worked/completion example pairs would be an effective strategy for fostering meaningful learning. The question is, however, which of these additional activities is best for improving learning: providing self-explanations, making concept maps or studying model concept maps?

### Hypotheses

Self-explanation is expected to be the best activity for promoting problem-solving skills in learners studying actual, complex cases. Evidence suggests that self-explanation is an efficient activity for promoting transfer [64,73]. Self-explanation induces cognitive processes that are geared more toward understanding the learning material [63] than organizing the information as is the case with concept mapping. Moreover, one can expect that concept mapping would be less effective than self-explanation for fostering problem-solving skills. That is because learners engaged in concept mapping need to dedicate a large part of their working memory to manipulating and organizing concepts and links in order to produce meaningful maps [77,80,81] at the expense of learning how to solve problems. However, one should notice that some evidence in health profession education shows that concept mapping can lead to significant improvements in understanding and problem-solving when compared to traditional teaching methods [54,92,93]. Concept map study is thought to be the best activity for promoting conceptual knowledge performance. A large body of evidence shows the potential of concept map study for improving knowledge recall [86-89], although its effects for improving transfer are not well substantiated [83]. However, one should take into account that concept map study might have other potential benefits because it is expected that it will impose lower cognitive load than self-explanation and concept mapping. Parallel to how example study reduces cognitive load compared to problem solving, learners engaged in concept map study do not have to invest effort in searching for the answer to a question or the connection between concepts, but rather, devote all available working memory resources to studying the connections between concepts. As such, concept map study can be expected to have a beneficial effect on cognitive load and learning. These hypotheses will be tested by comparing effort investment (an indicator of cognitive load) and learning outcomes among novice students who study worked/completion example pairs and additionally engage in self-explaining, concept mapping, or concept map study. Since clinical reasoning performance is context-dependent, it is relevant to present the domain of expertise in which this study will test these hypotheses: physiotherapy intervention knowledge.

### Clinical reasoning in the physiotherapy domain

As in other health professions, clinical reasoning in the physiotherapy domain involves cognitive processes such as pattern recognition and hypothetico-deductive reasoning to solve ill-structured clinical problems [94,95]. In this study, the field of knowledge will be the use of electrophysical agents in physiotherapy to treat patients with physical impairments. These modalities use thermal, acoustic, electrical and electromagnetic energy forms such as ultrasound therapy, therapeutic electrical currents, and low-level laser therapy that are often used by physiotherapists to treat patients with musculoskeletal, orthopaedic and neurological impairments [96]. The process-oriented worked examples designed, show the complete decision-making process involved in the selection of the optimal intervention among these agents. It is possible to present this complete decision-making process in the examples, because it relies on the selection of the best intervention among a restricted number of possibilities (i.e., electrophysical agent modalities). In the physiotherapy domain, problem-solving tasks that involve decision-making processes can be modeled by the following sequence of actions: problem recognition, problem definition, problem analysis, data management, solution development, solution implementation and outcome evaluation [97,98]. In this model, solution development includes data analysis and solution selection, whereas solution implementation refers to applying the solution to problem [97]. Process-oriented worked examples include the rationale for these steps (or actions) in order to help learners achieve meaningful learning on how to solve new problems in physiotherapy using electrophysical agents.

When considering the assessment of clinical-decision making, one should consider assessing both conceptual and procedural knowledge. This is because conceptual and procedural knowledge may not be closely related to one another [56,99,100], but can both influence clinical decision-making skills [56,59]. Moreover, in the context of this study, it is expected that conceptual knowledge and procedural knowledge will be differently influenced by integrative activities involving self-explanation, concept mapping, or concept map study [83]. Conceptual knowledge can be assessed using multiple-choice questions [101,102]. The assessment of procedural knowledge can be a challenge because it integrates many components, including tacit knowledge. Procedural knowledge can be evaluated using problem-solving tasks that allow one to consider strategic and teleological knowledge as well as heuristics that might influence decision making [56]. Meaningful learning will be evaluated by learners’ ability to solve new clinical problems that have the same structure but different surface features than the problems studied in examples (near transfer). Mental effort, an indicator of experienced cognitive load, will be measured as well to the efficiency of learning activities in terms of learning processes and learning outcomes in light of the cognitive load theory [103].

### Objectives

The first objective of this study was to compare the effects of engaging in self-explanation, concept mapping or model concept map study after studying/completing case-based worked and completion examples, on novice physiotherapy students’ conceptual knowledge and problem-solving performance. The second objective was to compare the cognitive load associated with these different instructional conditions.

## Method

### Participants and design

Ninety-one second-year physiotherapy students from Université de Montréal (Canada) participated in the study for course credits (mean age ± SD: 23.6 ± 4.4 years; 26 men; 65 women). All participants gave their informed consent to the study. The ethics committee of Université de Montréal’s Faculty of Medicine approved the study. Students at this stage of training have very little experience in selecting optimal physiotherapy interventions that use electrophysical agents. These electrophysical agents include various therapeutic electrical currents, ultrasound therapy and low-level laser therapy used by physiotherapists to treat patients with physical impairments that can result in the presence of pain, inflammation, weakness or functional deficits. All participants attended a three-hour instructional course on concept mapping using the free software IHMC Cmap tools available online (http://cmap.ihmc.us/) as part of their training. One week before the study, all students participated in a two-hour session on the basic principles of clinical reasoning underlying the selection of interventions with electrophysical agents in physiotherapy. Students were randomized into three groups and all studied worked/completion example pairs but additionally engaged in: self-explaining, concept mapping, or model concept map study. The study took place during a single, three-hour session at the participants’ university. This session was divided into three parts: a pre-test, a learning session and a post-test. Participants individually completed all the tests and learning activities in a digital learning environment. The learning session consisted of studying three pairs of worked/completion examples, with each pair of examples followed by an integrative activity. In the self-explanation condition, participants were instructed on the potential benefits of self-explanation for fostering learning, and were encouraged to do their best when answering self-explanation prompts (i.e., questions that aimed to engage them in explaining the previously studied examples). In the concept map completion condition, participants were instructed on potential benefits of concept mapping for fostering learning, and were instructed to do their best to complete a concept map in which main concepts were already presented, by adding and organizing concepts and generate crosslinks between concepts in order to represent the decision-making principles underlying previously studied examples. In the concept map study condition, participants were instructed on potential benefits of concept map study for fostering learning, and were instructed to do their best to study a complete model concept map showing decision-making principles presented in previously studied examples.

### Materials

#### Worked examples and completion examples

Six examples (three worked examples and three completion examples) were used in the present study. They presented clinical cases of patients admitted to physiotherapy for a physical impairment. Examples were written by incorporating the information collected from semi-structured interviews with four experienced physiotherapists who often use electrophysical agents in their day-to-day clinical practice. All interviews were audio-recorded and conducted by a physiotherapist from the research team (AH). The physiotherapists interviewed were aware that the information collected would be used to design instructional activities but were unaware of the exact activities or how they would be designed and used. During these interviews, the physiotherapists were asked to provide examples of clinical cases often encountered in their clinical practice for which the use of electrophysical agents is beneficial. For each case provided, they were instructed to specify the optimal electrophysical agent they chose, its parameters of application, the reasons for choosing that particular agent and why other electrophysical agents were less suitable for the patient’s problem. Clinical cases related to pain, inflammation and muscular weakness were most often cited as responsive to electrophysical agents. The electrophysical agents most often mentioned were superficial heat, cryotherapy, analgesic therapeutic currents, ultrasound therapy, neuromuscular electrical stimulation, low-level laser therapy and therapeutic iontophoresis. The interviewer drafted three pairs of worked and completion examples, pertaining to the management of pain, inflammation and weakness. The written examples were then revised by two other experienced physiotherapists to ensure their practicality, accuracy, clinical reasoning coherence and clarity.

The worked examples were designed to present the actions taken by a physiotherapist to manage a clinical problem using electrophysical agents from problem recognition to solution development [97]. Each written worked example (see Additional file 1) described a) the clinical problem of a patient in consultation with a physiotherapist; b) the physiotherapist’s detailed clinical reasoning, raising several hypotheses about potential electrophysical agents and explaining why they were or were not suitable for the case; c) the electrophysical agent selected by the physiotherapist; d) the parameters of application of the electrophysical agent chosen (e.g., intensity, frequency, electrode or probe placement, duration of treatment); and e) the key elements of the clinical case and context justifying the intervention selected. Each worked example was paired with a consecutive completion example (see Additional file 2) that dealt with the same type of problem (pain, inflammation or weakness) but in a different patient situation. The completion examples contained the same information as the worked examples, except for the fact that the final steps for solving the problem (steps c, d, and e) were left blank for the student to complete. The completion examples ended with the following questions: 1) “What is the most appropriate electrotherapy intervention for this case?” 2) “What are the optimal adjustment parameters for the electrophysical agent selected?” and 3) “What key characteristics of the case justify the intervention selected?”

#### Self-explanation sheets

For the self-explanation condition, three sheets were created, one for each worked/completion example pair (pain, inflammation and weakness). These sheets presented a question to prompt self-explanation about the worked/completion examples just studied and contained a text box with enough space for students to write out their explanation. For example, the question on the self-explanation sheet for pain read as follows: “Considering the worked/completion example that you just studied, can you explain the underlying principles for choosing electrophysical agents to treat a patient with any pain-related problem?”

#### Concept maps

For the concept map completion condition, the first author created three incomplete concept maps using the free software IHMC Cmap tools available online (http://cmap.ihmc.us/). These incomplete maps only presented concepts in boxes with rounded edges but did not present the links to explain the relationships between the concepts. The presented concepts were the main concepts of the decision-making processes used to select the electrophysical agents to treat problems associated with pain, inflammation or weakness, respectively. These concepts were similar to those provided in the worked and completion examples and were selected because of their relevance with respect to answering the specific question presented at the top of the map. The main question for the incomplete concept map relating to pain read as follows: “How do you choose a physical and electrophysical agent to treat a patient suffering from pain?” (see Additional file 3). The concepts presented were arranged to facilitate handling and organization into a complete concept map. These maps allowed students to generate more concepts, copy/paste concepts already shown, as well as generate and label crosslinks between the concepts and their relationships.

For the model concept map study condition, the first author created three model concept maps using the same software as for the incomplete maps. These maps presented visual diagrams of the decision-making processes used to select the electrophysical agents to treat problems associated with pain, inflammation or weakness, respectively. The model concept maps incorporated the information provided by the worked and completion examples. Each concept map was developed to answer a specific question, which was presented at the top of the map. The main question for the concept map relating to pain read as follows: “How do you choose a physical and electrophysical agent to treat a patient suffering from pain?” (see Additional file 4). The concept maps were drafted using a hierarchical technique with the most general concepts at the top and the more specific sub-concepts presented at the bottom. Concepts were presented in boxes with rounded edges and were connected by concept links. These links were presented by unidirectional arrow lines with a propositional statement to specify the nature of the relationships between the concepts.

#### Test tasks

A pre-test and a post-test evaluated students’ knowledge about the selection of physiotherapy interventions with electrophysical agents before and after the learning session, respectively. Each test evaluated conceptual knowledge and problem-solving skill. Both tests had the same format but presented different questions and tasks. Conceptual knowledge was measured with six multiple-choice questions with one correct answer (see Additional file 5). The maximum score was twelve points. Reliability of the conceptual knowledge pre-test and post-test was sufficient for group comparison (α Cronbach = 0.61 and 0.62, respectively). Problem-solving skill was measured with four tasks, consisting of a short case scenario and the following questions for students to answer: 1) “What is the most appropriate electrotherapy intervention for this case?” (1 point); 2) “What are the optimal adjustment parameters for the electrophysical agent selected?” (2 points); and 3) “What key characteristics of the case justify the intervention selected?” (2 points) (see Additional file 6). The maximum score was 20 points. Reliability of the problem-solving pre-test and post-test was sufficient for group comparison (α Cronbach = 0.63 and 0.71, respectively). These tasks aimed to assess learners’ capacity to carry out specific actions when attempting to solve a physiotherapy problem. These actions were: a) *solution development* by data analysis (i.e., extract the key features of the problem) and solution selection (i.e., select the most appropriate electrophysical agent), and b) *solution implementation* by applying a solution to a problem (i.e., parameterizing the selected electrophysical agent) [97]. Performance on the post-test problem-solving tasks was used to measure near transfer performance. Therefore, the problems presented in the post-test had different surface features but a similar structure to the problems that were presented in the examples and completion problems. In order to determine the reliability of the scoring procedure, two independent raters unaware of the experimental procedure, scored approximately 10% of the data (i.e., 9 participants). The intra-class correlations coefficients (ICC) were 0.97 for the pre-test and 0.96 for the post-test. Because of these high correlations, the rest of the scoring was done by only one of the raters and this rater’s scores were used in the analysis.

#### Mental effort rating scale

The mental effort invested in the learning activities and the post-test was evaluated after each of the tasks with a nine-point subjective rating scale ranging from 1, which represented very, very low mental effort to 9, which represented very, very high mental effort. This scale, which was developed by Paas, (1992) is widely used in educational research [4,103]. Mental effort provides an indication of the cognitive load imposed by the task [49,104].

### Learning environment

The pre-test, post-test, worked/completion examples pairs, and experimental interventions (self-explanation prompts, concept map tools and model concept maps), and mental effort rating scales were presented to the participants within the Université de Montréal digital learning environment. This environment was designed based on the e-learning Moodle platform (version 2.5). All participants were familiar with this learning environment given that they had been using it regularly for over a year in other courses. Answers to tests and mental effort rating scales were entered into the learning environment and analyzed *a posteriori*.

### Experimental procedure

**Figure 1:**
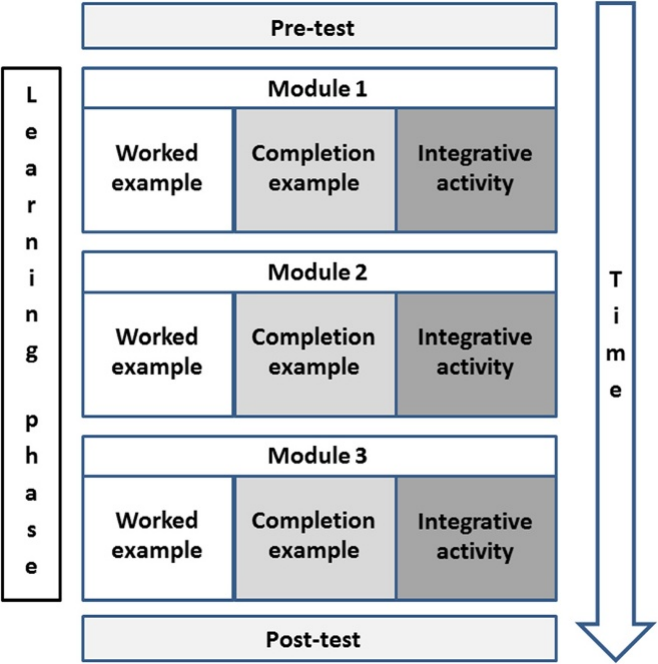
**Time-course of the study.** Students took part in a pre-test, a learning phase and a post-test. The learning phase consisted of three modules on pain, inflammation and weakness, respectively, presented in random order for each student. Each module consisted of a worked example followed by a completion example and an integrative activity on the same topic. Students were randomized into the self-explanation, self-mapping or concept map study as an integrative activity.

Figure 1 presents the experimental session that lasted three hours and twenty minutes, including rest breaks. After a 5-minute introduction on the procedure of the study, students had a maximum of thirty-five minutes to complete the pre-test and the associated mental effort rating which was followed by a 5-minute break. Participants then had to participate in three learning modules presented in random order, on pain, inflammation and weakness. Each module consisted of a worked example, a completion example and an additional integrative learning activity (self-explaining, concept map completion or model concept map study, depending on the assigned condition). Each worked example was presented for eight minutes with the following instructions: “You have 8 minutes to carefully study the following example. Later, you will have to complete a similar example by yourself.” Each completion example was presented for ten minutes with the following instructions: “You have 10 minutes to carefully study the following example and answer the questions at the end.” Students could refer back to the previous worked example while completing the completion example as many times as they wished. After each pair of worked/completion examples, students had 15 minutes to participate in the integrative activity, depending on their randomly assigned condition. This activity involved 1) self-explaining the principles underlying the examples (self-explanation condition); 2) completing a concept map representing these principles from a sheet on which key concepts were presented (concept mapping condition); or 3) studying a model concept map presenting these principles (concept map study condition). The participants then had to rate the mental effort they invested while studying the module. Students took a 5-minute break after completing each module. Then they had thirty-five minutes to complete the post-test, after which they again rated the mental effort invested in the test. The duration of each activity was pilot-tested with students from a previous cohort who did not participate in the present study.

### Data analysis

Problem-solving performance on the pre- and post-test was scored using the same procedure. For each question, a pre-determined checklist of acceptable responses and a scoring grid associated with these responses was used. This checklist was drafted by the physiotherapist who conducted the interviews with the expert physiotherapists, and was further reviewed and slightly revised by an experienced physiotherapy teacher. To verify random assignment, ANOVAs were conducted to compare participants’ age and conceptual and problem-solving knowledge on the pre-test among the three conditions. To assess whether conceptual knowledge and problem-solving performance were correlated between the pre-test and the post-test, Pearson correlations were performed. To assess differences in learning outcomes of the three conditions, analyses of covariance (ANCOVAs) were conducted on the conceptual knowledge and problem-solving performance scores on the post-test, with pre-test conceptual knowledge and problem-solving performance as covariates, respectively. Lastly, to explore whether the conditions differentially affected cognitive load, an ANOVA on mental effort invested in the learning phase and on the post-test was conducted. When univariate effects were significant, Tukey post-hoc HSD analyses were performed. Spearman rank correlations were performed to assess correlation between the mental effort invested and performance for the post-test. Effect sizes were measured using partial η^2^ with .01, .06 and > .14 considered as weak, medium and large effect sizes, respectively [105]. P values ≤ .05 were considered significant. Statistical analyses were performed using the Statistical Package for Social Science software (SPSS) (version 19 for Windows).

## Results

There were no significant differences between the self-explanation, concept map completion and model concept map study conditions in age (F (2, 88) = .683; P = .508), in conceptual knowledge (F (2, 88) = .028; P = .972), or problem-solving performance (F (2, 88) = 2.481; P = .089) on the pre-test (see Table 1).

**Table 1:** **Mean performance (SD) on the pre-test and post-test expressed as a percentage of the maximum score**

		a) Self-explanation	b) Concept mapping	c) Concept map study	Tukey post-hoc HSD		
					P _ a-b _	P _ a-c _	P _ b-c _
**Pre-test** (% max. score)	Concept. knowledge	48.89 (16.91)	49.44 (17.77)	50.00 (20.18)			
	Problem-solving	41.27 (9.30)	35.37 (9.95)	37.95 (11.44)			
**Post-test** (% max. score)	Concept. knowledge	52.78 (17.00)	52.22 (21.77)	68.82 (21.83)	.994	**.008**	**.006**
	Problem-solving	63.16 (16.02)	50.98 (13.60)	53.34 (16.37)	**.008**	**.038**	.821

Concept. knowledge: Conceptual knowledge. **P**_**a -b,**_**P**_**a -c,**_**P**_**b -c**_: P values for comparisons between self-explanation and concept mapping, self-explanation versus concept map study and concept-mapping versus concept map study, respectively. **P** values < .05 are in bold.

The conceptual knowledge on the post-test was correlated with conceptual knowledge on the pre-test (r = .21, *P* = .047). Similarly, problem-solving performance was correlated between the post-test and the pre-test (r = .24, *P* = .020). Because of these correlations, conceptual and problem-solving knowledge on the pre-test were considered to be covariates when assessing the effect of the condition on conceptual knowledge and problem-solving performance on the post-test. The ANCOVA with conceptual knowledge as the covariate on the pre-test showed a significant medium condition effect on conceptual knowledge on the post-test (F (2, 87) = 6.656, *P* = .002, η2 = .133) and the ANCOVA with problem-solving performance on the pre-test as the covariate showed a medium condition effect on problem-solving performance (F (2, 87) = 3.984, *P* = .022, η2 = .084). Conceptual knowledge on the post-test in the model concept map study condition was higher than in the self-explanation (Tukey HSD, *P* = .008) and concept map completion (*P* = .006) conditions (see Table 1). Conceptual knowledge was not significantly different between the self-explanation and concept map completion conditions (*P* = .994). Problem-solving performance on the post-test in the self-explanation condition was higher than in the model concept map study (*P* = .038) and the concept map completion (*P* = .008) conditions. Problem-solving performance on the post-test was not significantly different between the concept map completion and model concept map study conditions (*P* = .821).

There was a large main learning condition effect on the mean mental effort invested during the learning activity (ANOVA, F (2, 88) = 7.68, *P* = .001; η2 = .149). The mean mental effort invested in the model concept map study condition was lower than in the concept map completion (Tukey HSD, *P* = .001) and self-explanation conditions (*P* = .015) (see Table 2). There was no significant difference in the mental effort invested between the concept map completion and self-explanation conditions (*P* = .649).

**Table 2:** **Mean mental effort invested (SD) in the learning activity and in the post-test for the three learning conditions**

		a) Self-explanation	b) Concept mapping	c) Concept map study	Tukey post-hoc HSD		
					P _ a-b _	P _ a-c _	P _ b-c _
**Learning phase**	Mental effort (max = 9)	6.70 (0.86)	6.92 (0.79)	6.02 (1.13)	.649	**.015**	**.001**
**Post-test**	Mental effort (max = 9)	5.90 (0.92)	7.07 (0.83)	6.52 (1.03)	**.001**	**.030**	.060

**P**_**a -b,**_**P**_**a -c,**_**P**_**b -c**_: P values for comparisons between self-explanation and concept mapping, self-explanation versus concept map study and concept-mapping versus concept map study, respectively. **P** values < .05 are in bold.

There was a significantly large condition effect on the mental effort invested in the post-test (F (2, 88) = 11.785, *P* < .001, η2 = .211). The mental effort invested in the post-test for the self-explanation condition was lower than in the concept map completion (*P* < .001) and model concept map study (*P* = .03) conditions (see Table 2). The mental effort invested in the post-test was not significantly different between the concept map completion and model concept map study conditions (*P* = .060). The mental effort invested in the post-test was negatively correlated with global performance (conceptual knowledge and problem-solving) on the post-test in the self-explanation (r = −.527, *P* = .003), concept map completion (r = −.482, *P* = .007) and model concept map study (r = −.665, *P* < .001) conditions.

## Discussion

In this study, learners studying worked/completion examples pairs were additionally engaged in self-explanation, concept map completion or model concept map study to deepen their understanding and knowledge of physiotherapy intervention and to promote meaningful learning. This study aimed to investigate which of these additional activities would be best for improving conceptual knowledge and problem-solving skills. It was expected that the self-explanation and concept map study activities would be most effective in fostering problem-solving skills and conceptual knowledge, respectively.

Consistent with our hypothesis, learners who self-explained outperformed those who engaged in concept map completion and concept map study on post-test problem solving, and students who studied concept maps outperformed those in the other two conditions on the conceptual knowledge post-test. During the post-test, students in the self-explanation condition had to invest less mental effort than those in the concept map study and concept map completion conditions. This suggests that the cognitive schemas that students in the self-explanation condition acquired (i.e., learning *outcomes*) were more efficient [103]: they allowed for better problem solving with less investment of mental effort on the test. When considering efficiency in terms of the learning *process*, the concept map study condition was most efficient: students in this condition invested less mental effort than those in the other two conditions, while acquiring more conceptual knowledge.

The increased problem-solving performance among students who self-explained demonstrates that self-explanation leads to a better *understanding* of the principles behind the solution steps demonstrated in the worked examples (i.e., understanding the how and why of these steps). Self-explanation allows them to perform more efficiently (i.e., with less effort) on novel problems. These results are in accordance with previous studies showing the effectiveness of self-explanation in fostering transfer of learning of problem-solving skills among learners studying worked out examples [35,66]. These studies compared learning with worked examples with and without prompts to promote self-explanation. Moreover, some evidence suggests that self-explanation promotes more learning than instructional explanation [106]. However, integrating instructional explanations into learning via self-explanations has a positive effect on learning [71]. An interesting aspect is the fact that learners who self-explained benefitted from this activity without any prior training in self-explanation. This shows potential for educational practice, as only short instruction on why self-explaining is useful, combined with prompting self-explanations, fostered meaningful learning. One can hypothesize that prior self-explanation training might promote learning even further. To the best of our knowledge, the present study is the first to compare the effects of providing self-explanation to other additional integrative learning activities that promote meaningful learning in example-based learning with worked examples.

Self-explaining did not foster conceptual knowledge; however; conceptual knowledge was improved by concept map study. Since these maps provided a meaningful outline of the concepts pertaining to physiotherapy intervention knowledge, it might have helped students to incorporate in-depth concepts into their schemas compared to the other conditions, where this information was lacking or had to be generated by the students. In this respect, one should note that complete and incomplete concept maps provided more information than self-explanation. Therefore, care should be taken when interpreting these results since rote memorization of the information presented in maps might have accounted for the higher conceptual knowledge in the concept map study condition compared to the self-explanation condition. Studying these maps even lowered the cognitive load compared to other conditions. This is consistent with previous findings that showed the potential of concept map study in improving recall performance [85-89]. Concept maps may facilitate learning by summarizing the information [83]. When compared to text summaries, concept maps can be reviewed more quickly, allowing learners to review the information several times within a fixed time period [83]. By presenting models, concepts and links in an organized way, concept maps chunk the information, allow learners to manage and assimilate a large amount of information [82]. Our results are also consistent with a previous review reporting more evidence on the effects of map study in improving recall than transfer performance [83]. This suggests that conceptual knowledge may be necessary, but not sufficient to guide problem solving, for which strategy knowledge (i.e., understanding how to handle a task and why one approach is more effective for a certain problem type than another) is crucial. By directly comparing these conditions, the present study provided further insight on the potential of concept map study to foster conceptual knowledge when compared to self-explanation and concept mapping. Moreover, this raises the question as to whether, in example-based learning, the combination of self-explanation and concept map study could promote learning more than the addition of each of these integrative activities alone. In light of present results, one can hypothesize that additionally providing self-explanations of principles presented in examples followed by the study of a model concept map presenting these principles might foster both problem-solving skills and conceptual knowledge. Future studies should address the effects of combining these integrative learning activities on clinical reasoning skills of learners engaged in example-based learning.

Concept mapping was not very effective for acquiring problem-solving or conceptual knowledge. One main difference with concept map study is that it involves several other cognitive processes: concept mapping involves the assessment of concept inclusivity and specificity, the incorporation of lower-order concepts under high-order concepts and integrative reconciliation to establish links between differentiated concepts [77,80,81]. This in itself could have distracted learners from the main learning task, that is, it may have put a significant extraneous (i.e., ineffective) load on working memory, at the expense of learning. Notably, the self-explanation activity generated a comparable level of mental effort but led to better learning compared to the concept mapping condition. It was expected that the self-explanation condition would generate a substantial amount of cognitive load when considering all the cognitive processes involved in this activity. However, these processes would be expected and were relevant for learning. Moreover, it is worth noting that only a single measure of cognitive load, that is, *self-reported mental effort invested* [107], was used in this study. This measure can reliably assess experienced cognitive load, and is often used in cognitive load research because it provides relevant information in combination with learning outcome measures [104,108]. One drawback of this measure, however, is that it does not pinpoint which processes imposed the reported cognitive load. Attempts have been made to distinguish this among the different types of load [109], although there is not enough conclusive empirical evidence to accurately assess the different types of load. Other options for cognitive load measurement that would not rely on subjective ratings, would be to use objective and continuous measures of cognitive load, such as response times during dual-task performance [108], or neuroimaging and electrophysiological techniques [108,110].

Another technique that could provide more insight into the processes that impose cognitive load would be to use eye-tracking measures while students study in the learning environment. Process-tracing data from log files, verbal reports (i.e., think-aloud), or eye-tracking would provide more insight into students’ cognitive processes, interactions within the learning environment, and their level of engagement during learning activities which we do not have at the moment. For instance, think-aloud protocols could have provided information on other explanations that students may have considered before they typed in their answer. All three types of data processing could have allowed us to investigate how often learners switched between the worked and completion examples (which they were allowed to do) and whether the number of switches is positively or negatively related to their learning outcomes. Also, in studies such as the present one where learners received three worked/completion example pairs, eye tracking might reveal evidence of skill acquisition during the learning phase. For instance, a recent eye-tracking study of example-based learning in a digital learning environment [111] showed that there were differences between novice and advanced learners in what material they paid attention to. So although these techniques are costly in terms of the time required for data collection and data analysis, now that the effects on learning have been established, it would be interesting to use process-tracing techniques in future studies to investigate the mechanisms through which these effects come about, in more detail.

In this study, meaningful learning of intervention knowledge, that is clinical reasoning in the selection of intervention with electrophysical agents, was assessed by problem-solving skills. Moreover, conceptual knowledge was also assessed because it can influence clinical reasoning. Among the limitations of the study, one should note that the conceptual knowledge and problem-solving test involved different types of retrieval. Since the open-ended questions of problem-solving test involved more active and in depth recall than the multiple-choice questions of the conceptual knowledge test, results could possibly be, at least partly, attributed to a difference in the types of recall fostered by learning activities. When considering present results, one can hypothesize that self-explanation and concept map completion could have fostered in-depth and superficial retrieval, respectively. A limitation related to the problem-solving test is the fact that it assessed only one subset of problem-solving skills. Two problem-solving actions were measured, that is, solution development and solution implementation. When considering May and Newman’s model, some important problem-solving actions such as problem analysis and data management were not specifically assessed [97]. Moreover, the study did not specifically assess the strategic and teleological knowledge that contributes to intervention knowledge and problem-solving skills. Future studies should investigate which of the different types of knowledge involved in problem-solving are fostered by example-based learning.

Some limitations of the study are related to its external validity and the extent to which these results would apply in other domains and real-world educational settings. One should note that results are in accordance with those found in various domains showing that self-explanation and concept map study are useful strategies to foster problem-solving skills and conceptual knowledge, respectively [65,83]. This is an argument in favor of the hypothesis that similar results are likely to be found in other domains. In this study, learning activities were used in a single learning session and for a limited time of exposure. These experimental conditions are not representative of real-world learning environments, in which learners are often engaged in activities with more repetitive exposure and for longer periods of time than in the present study. However, given the benefits gained in a short learning session, one can expect that repeated and longer exposure to these activities would have produced greater overall learning effects. For instance, repeated self-explanation, once a week for three weeks, has been shown to improve retention and transfer in medical students [73]. Most studies using concept mapping as a learning tool have assessed their effects when concept mapping is used over a certain period of time [77]. For example, creating a concept map over the course of a semester was found to improve critical thinking in nurses [77]. Similarly, studying an expert concept map over a one-week period improved knowledge among resident physicians [91]. It is possible that short-duration exposure to learning activities would have impacted concept mapping more than self-explanation or concept map study. It is possible that concept mapping will impose lower extraneous load and becomes more useful for learning once students gain prior knowledge, so over longer learning periods, concept mapping may become more effective. Future studies should compare these activities in conditions that are representative of real-world learning environments.

This study assessed near-transfer but not far-transfer of problem-solving. When considering example-based learning with worked examples, it can be expected that adding self-explanation, in particular, would promote far transfer [66]. However, other evidence suggests that studying model concept maps as advanced organizers can foster far transfer [112-114]. Although concept mapping can promote near transfer in health profession education [54,92,93], no evidence suggests that it can provide an advantage over other learning activities in fostering far transfer. Future studies should compare the effects of self-explanation, concept mapping and concept map study on the ability of learners to solve problems that are more varied than those studied here and to examine the effect that self-explanation has on post-tests that are delayed over a few days or weeks. Such studies would be relevant in assessing whether sustained learning occurs and would be more representative of real-world learning environments.

## Conclusions

Additional self-explanation after completing worked/completion example pairs produces better performance on immediate near-transfer than additional self-mapping or concept map study. Additional study of a model concept map after completing worked/completion example pairs produces better performance on an immediate conceptual knowledge test than additional self-explanation or self-mapping. Future studies should compare the long-term effects on performance on a far-transfer test of repeated self-explanation, self-mapping and concept map study of clinical reasoning skills.

## Additional files

Additional file 1: **Worked example on inflammation.** Worked example presenting the actions taken by a physiotherapist to manage a clinical problem associated with inflammation using electrophysical agents from problem recognition to solution development. Additional file 2: **Completion example on pain.** Completion example presenting the actions taken by a physiotherapist to manage a clinical problem associated with pain using electrophysical agents in which the final steps for solving the problem are left blank and replaced by questions for the student to answer. Additional file 3: **Incomplete concept map on pain.** Incomplete concept map presenting the main concepts of the decision-making processes used to select the electrophysical agents to treat problems associated with pain. TENS: Transcutaneous electrical nerve stimulation. Additional file 4: **Concept map on pain.** Concept map presenting the main concepts of the decision-making processes used to select the electrophysical agents to treat problems associated with pain. TENS: Transcutaneous electrical nerve stimulation; Diadynamic; Diadynamic current; US: Ultrasound therapy; LASER: Low level laser therapy. Additional file 5:
**Multiple-choice questions.**
Additional file 6:
**Questions of transfer tests.**
