# Supplementary material for: Example-based learning: comparing the effects of additionally providing three different integrative learning activities on physiotherapy intervention knowledge
Source: BMC Med Educ. 2015 Mar 7;15:37. doi: 10.1186/s12909-015-0308-3 (PMC4414367; doi:10.1186/s12909-015-0308-3)
Supplement: Additional file 1: — Worked example on inflammation. Worked example presenting the actions taken by a physiotherapist to manage a clinical problem associated with inflammation using electrophysical agents from problem recognition to solution development. [file 12909_2015_308_MOESM1_ESM.docx]

Additional fie 1

Worked example on inflammation

***A) Initial presentation of the clinical case***

*Michel B. is a 38-year-old, right-handed male and works as an orderly. He consults you for pain in his right shoulder that developed gradually since he switched work departments (new customers) one month ago. The pain is diffuse but he feels it more on the lateral side of the shoulder and it radiates to the right arm. Pain is felt upon elevation movements of the arm in flexion or abduction past 90° and is increased with lifting tasks. The pain wakes him up at night if he is in a lateral decubitus position (regardless of the side). He saw a doctor last week who diagnosed him with* ***rotator cuff tendinopathy*** *and prescribed him non-steroidal anti-inflammatory drugs (NSAIDs) and physiotherapy. The drugs provided relief for the first few days, but his pain continued to worsen until it became constant. He now has weakness in the* ***right arm*** *that affects his productivity at work.*

**B) Following the subjective (S) and objective (O) evaluations: Analysis (A)**

Physiotherapist’s rationale during his analysis of the situation: This patient probably has rotator cuff tendinopathy due to shoulder impingement syndrome resulting from a lack of scapular stability and scapulohumeral control. In terms of alteration in scapulohumeral rhythm, I notice weakness in flexion, abduction and external rotation of the right shoulder associated with pain and lack of coordination of the muscles that control the scapula. More specifically, based on the painful palpation site and a positive Empty Can Test, it seems that the right supraspinatus is the most affected and inflamed (subacute: x 1 month). My subjective and objective evaluations helped me eliminate the unlikely assumptions of a systemic problem, cervical neuropathy, capsulitis or biceps tendinopathy.

My PRIORITIZED list of problems associated with this case includes: 1) significant pain at the right shoulder; 2) decrease in active right shoulder movements; 3) weakness of the right rotator cuff muscles; 4) decreased control of stabilizing muscles of the scapula; 5) decreased function in ADL and at work; 6) patient lacks knowledge about his condition.

My physiotherapy diagnosis would be: Right shoulder impingement syndrome exacerbated by occupational factors and associated with dynamic instability of the right scapula, resulting in rotator cuff tendinopathy, which restricts participation in ADL and at work.

**Clinical reasoning behind the treatment plan (P):**

Physiotherapist’s rationale for treatment plan: Shoulder pain results from recurrent irritation of the rotator cuff muscles, especially the supraspinatus because of the repeated impingement of the tendon on the acromion during elevation movements of the right arm. This impingement results from mechanical imbalance at the shoulder due to a lack of control of the right stabilizing scapular muscles. To restore proper biomechanics, scapular stabilizing muscles should be trained with voluntary control EXERCISES and scapulohumeral rhythm should also be rehabilitated (evidence of long-term effects on pain).

In addition, one of the therapeutic aims will be to provide **LONG-TERM ANALGESIA THROUGH ANTI-INFLAMMATORY EFFECTS THAT SPECIFICALLY ADDRESS THE PAINFUL SITE.** Therefore, we should use **AN ADJUVANT ANALGESIC MODALITY FOLLOWING EXERCISE** that does not interfere with pain signals during exercise. The modalities to consider are: **Ice** (used more during acute condition, interferes with the effect of exercises, non-specific action, hinders local circulation). **Heat** (non-specific action, muscular structures already warmed up by exercises, tendon structures too deep). **Diadynamic** (non-specific action, little evidence). **TENS** (specific analgesia documented, specific effects but no effect on inflammation). **Interferential current** (same as TENS, but more diffuse analgesic effect on a larger area of tissue). **High voltage** (localized action, anti-inflammatory (polar effects), good action depth, little evidence). **Laser** (sufficient evidence for tendinopathy but the action depth is not sufficient (1cm)). **Ultrasound** (acceptable alternative because it does not irritate, local mechanical effects to clear the tendon of any debris and increase circulation, anti-inflammatory effects and sufficient action depth (5cm); however, if the damage is more muscular, the muscle will already be warmed up with exercise, but if it affects the tendon more, ultrasound should be considered with subacute parameters). **Continuous constant current** (Iontophoresis): best alternative because there is documented evidence to support the anti-inflammatory effects of a subacute condition with corticosteroids: Dexa (hydrocortisone); nevertheless, we must verify the patient’s level of irritation and the medical recommendation.

| **C) Intervention plan (P): Iontophoresis**  **D) E.g., with hydrocortisone acetate (0.5%); at a dose of 45-60 mA.min; 3-4 mA x 15 min.;**  **E) Pain resulting from active inflammation; subacute condition; structure’s depth; medical prescription required** |
| --- |
