# Supplementary material for: Example-based learning: comparing the effects of additionally providing three different integrative learning activities on physiotherapy intervention knowledge
Source: BMC Med Educ. 2015 Mar 7;15:37. doi: 10.1186/s12909-015-0308-3 (PMC4414367; doi:10.1186/s12909-015-0308-3)
Supplement: Additional file 2: — Completion example on pain. Completion example presenting the actions taken by a physiotherapist to manage a clinical problem associated with pain using electrophysical agents in which the final steps for solving the problem are left blank and replaced by questions for the student to answer. [file 12909_2015_308_MOESM2_ESM.docx]

Additional fie 2

Completion example on pain

***A) Initial presentation of the clinical case***

*Mr. Clark is 69-year-old retired male. He is referred to physiotherapy for* ***chronic low back pain radiating down the right leg to the foot.*** *It is residual pain from lumbar L3-L5 decompression surgery performed to treat spinal stenosis 14 months ago. The pain, which feels somewhat like electric shocks, is exacerbated in a standing position and makes walking impossible over medium distances (> 30 m). Walking with the support of both hands (crutches) reduces pain and increases walking distance.* ***Mr. Clark complains of being more and more deconditioned.*** *He takes opioid analgesic (codeine) and anti-inflammatory drugs, which are becoming less effective every month.*

**B) Following the subjective (S) and objective (O) evaluations: Analysis (A)**

Physiotherapist’s rationale during his analysis of the situation: This patient probably suffers from severe chronic low back pain with spinal stenosis and radiculopathy (L5), which persisted after his spinal decompression surgery. The patient’s clinical data also suggests the presence of a central sensitization phenomenon of pain perception. Mr. Clark reports of pain at rest that increases to 8.5/10. He is extremely anxious about his pain and displays a lot of apprehension when we touch him or ask him to move. All active movements involving the trunk or right lower limb are very painful. Moreover, the patient is not responding well to opioids and anti-inflammatory drugs. Dysesthesias are also present at the right lower limb. My subjective and objective evaluations helped me eliminate the unlikely assumptions of pyramidal syndrome, cauda equina syndrome (Horse’s tail compression) or an orthopedic injury to the lower limb.

My PRIORITIZED list of problems associated with this case includes: 1) significant low back pain and right lower limb pain (L5 area); 2) anxiety regarding pain; 3) general decreased mobility; 4) deconditioning; 5) decreased function during ADL and DADL; 6) patient lacks knowledge about his condition.

My physiotherapy diagnosis would be: chronic low back pain with right radicular L5 syndrome, which has persisted since lumbar decompression surgery, with potential central sensitization that limits general mobility and walking and restricts participation in ADL and DADL.

**Clinical reasoning behind the treatment plan (P):**

Physiotherapist’s rationale for treatment plan: Low back pain probably resulting from mechanical damage that persisted following surgery, while right lower limb pain might be due to a damaged L5 root. The sensitization phenomenon probably reflects changes resulting from pain hypersensitivity due to central neuroplasticity. Thus, goal of therapy should be to mobilize the patient and rehabilitate his perception of pain. An effective analgesia must be provided **DURING** the **EXERCISES** during the therapy session in order for the patient to achieve optimal mobility.

**1) What is the most appropriate electrotherapy intervention for this case?**

**2) What are the optimal adjustment parameters for the electrophysical agent selected?**

**3) What key characteristics of the case justify the intervention selected?**
