# Supplementary figures and images for: Example-based learning: comparing the effects of additionally providing three different integrative learning activities on physiotherapy intervention knowledge
Source: BMC Med Educ. 2015 Mar 7;15:37. doi: 10.1186/s12909-015-0308-3 (PMC4414367; doi:10.1186/s12909-015-0308-3)

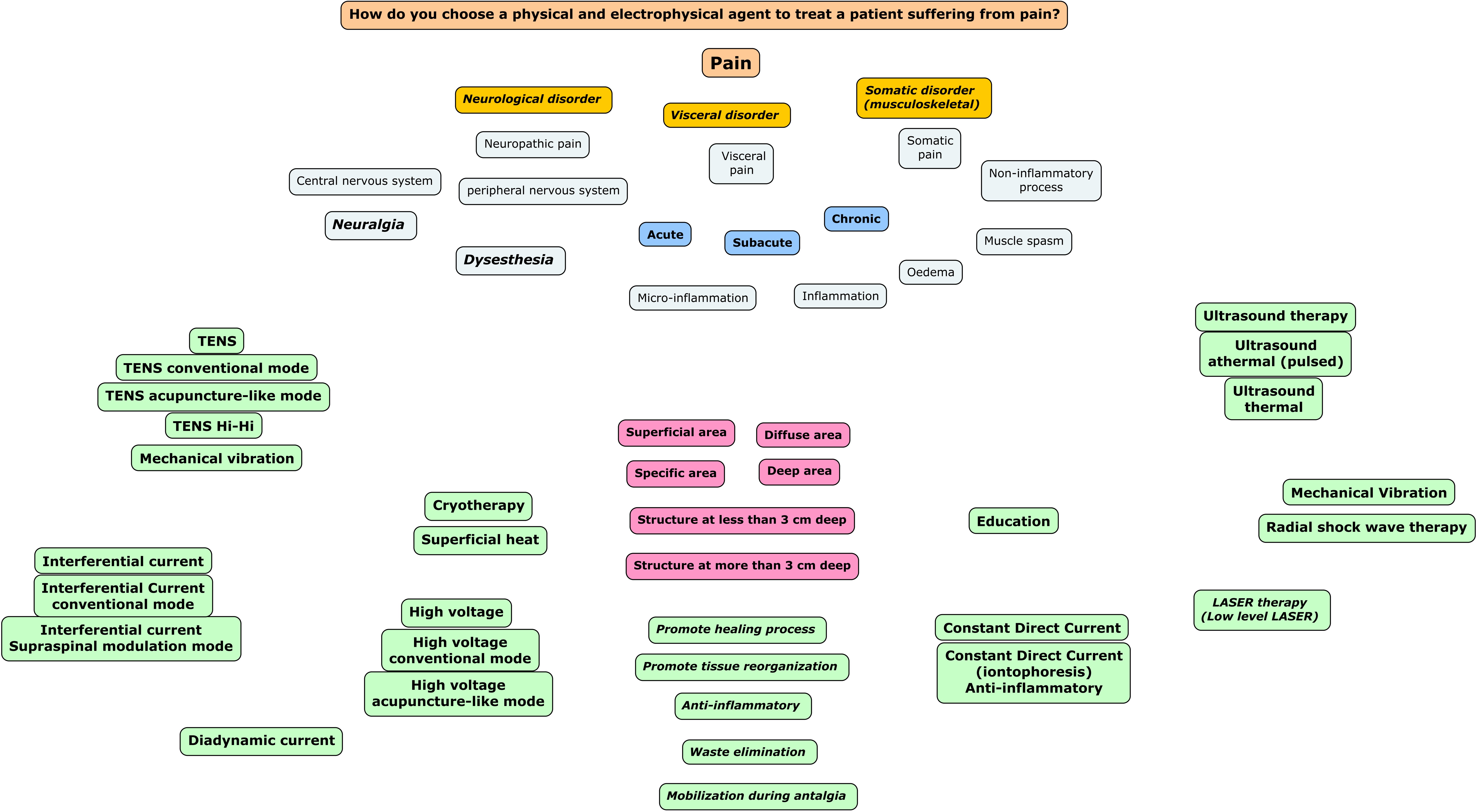

Supplement: Additional file 3: — Incomplete concept map on pain. Incomplete concept map presenting the main concepts of the decision-making processes used to select the electrophysical agents to treat problems associated with pain. TENS: Transcutaneous electrical nerve stimulation. [file 12909_2015_308_MOESM3_ESM.jpeg]
