# Supplementary material for: Example-based learning: comparing the effects of additionally providing three different integrative learning activities on physiotherapy intervention knowledge
Source: BMC Med Educ. 2015 Mar 7;15:37. doi: 10.1186/s12909-015-0308-3 (PMC4414367; doi:10.1186/s12909-015-0308-3)
Supplement: Additional file 5: — Multiple-choice questions. [file 12909_2015_308_MOESM5_ESM.docx]

Additional fie 5

Multiple-choice questions

Question A: When considering the theoretical therapeutic effects of cryotherapy and thermotherapy with superficial heat, one can state that:

1) The application of ice reduces the activity of neuromuscular spindles, which helps to diminish muscular spasms;

2) The application of ice helps increase the activation threshold of nociceptive afferents within cooled tissues;

3) Some physiological properties of tissue heating contribute to its anti-inflammatory effects;

4) Topical balms can be used in combination with superficial heat because balms and heating do not trigger the same antalgic mechanisms.

Choose one answer:

a) 1, 2 and 3 are correct

b) 1 and 3 are correct

c) 2 and 4 are correct

d) Only 4 is correct

e) All of the statements are correct

Question B: When selecting and combining physical agents and electrotherapeutic modalities, one should consider the following:

1) Conventional neuromuscular stimulation is more suited to motor control exercises than Russian current;

2) The application of ice involves more risk than superficial heating of the lower extremities among patients affected by peripheral vascular disorders;

3) There is more scientific evidence for the use of high voltage therapy compared to ultrasound therapy for improving pressure wound healing;

4) There is more evidence on the use of LASER therapy for lateral epicondylitis than for rotator cuff tendonitis.

Choose one answer:

a) 1, 2 and 3 are correct

b) 1 and 3 are correct

c) 2 and 4 are correct

d) Only 4 is correct

e) All of the statements are correct
