# Supplementary material for: Example-based learning: comparing the effects of additionally providing three different integrative learning activities on physiotherapy intervention knowledge
Source: BMC Med Educ. 2015 Mar 7;15:37. doi: 10.1186/s12909-015-0308-3 (PMC4414367; doi:10.1186/s12909-015-0308-3)
Supplement: Additional file 6: — Questions of transfer tests. [file 12909_2015_308_MOESM6_ESM.docx]

Additional fie 6

Questions of transfer tests

**Question A:** A young athletic patient arrives at your clinic with a grade II sprain of the lateral collateral ligament (anterior talo-fibular) of the right ankle which occurred two days ago after an inversion trauma. He arrives on crutches. The ankle is swollen and unable to bear weight. He reports pain in the ankle, under the lateral malleolus. The pain increases with weight bearing and with inversion.

Following your assessment, the main problems identified are as follows (in order of priority): 1) pain (7/10) under the lateral malleolus with weight bearing and with inversion; 2) interstitial, hard, edema at the right ankle; 3) decreased weight bearing on the right lower extremity; 4) decreased ROM in all movements of the right ankle; 5) loss of strength of the right ankle; 6) limping while walking.

**1) What is the most appropriate electrotherapy intervention for this case?**

**2) What are the optimal adjustment parameters for the electrophysical agent selected?**

**3) What key characteristics of the case justify the intervention selected?**

**Question B:** A 46-year-old lady enters your clinic with a medical reference for a lumbar herniated disc at L5, with S1 radiculopathy. Her symptoms appeared four days ago. Oral non-steroidal anti-inflammatory drugs appear to relieve the pain but the lady does not feel any additional relief when taking codeine (opiate) orally.

Following your assessment, the main problems you identify are as follows (in order of priority): 1) severe lower back pain (P1); 2) moderate pain (P2) on the trajectory of the S1 root of the right lower limb; 3) overall decreased mobility; 4) decreased ROM in the lumbar spine and in the right lower limb; 5) decreased walking autonomy with crutches; 6) limited work performance when she must remain seated at the computer.

**1) What is the most appropriate electrotherapy intervention for this case?**

**2) What are the optimal adjustment parameters for the electrophysical agent selected?**

**3) What key characteristics of the case justify the intervention selected?**
